# Phase Separation and Fibrillization of Human Annexin A7 Are Mediated by Its Proline-Rich Domain

**DOI:** 10.1021/acs.biochem.3c00349

**Published:** 2023-10-03

**Authors:** Chenrong Yu, Spencer L. Nelson, Georg Meisl, Rodolfo Ghirlando, Lalit Deshmukh

**Affiliations:** † Department of Chemistry and Biochemistry, University of California San Diego, La Jolla, California 92093, United States; ‡ Department of Chemistry, University of Cambridge, Cambridge CB2 1EW, U.K.; § Laboratory of Molecular Biology, National Institute of Diabetes and Digestive and Kidney Diseases, National Institutes of Health, Bethesda, Maryland 20892, United States

## Abstract

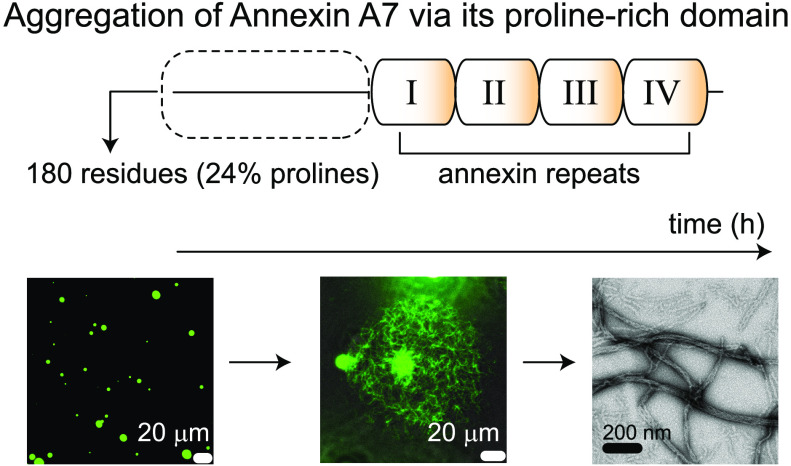

Human annexin A7, a calcium- and phospholipid-binding protein, governs calcium homeostasis, plasma membrane repair, apoptosis, and tumor progression. A7 contains an N-terminal proline-rich domain (PRD; 180 residues, ∼24% prolines) that determines its functional specificity. Using microscopy and dye-binding assays, we show that recombinant A7 and its isolated PRD spontaneously phase separate into spherical condensates, which subsequently transform into β-sheet-rich fibrils. We demonstrate that fibrillization of A7-PRD proceeds via primary nucleation and fibril-catalyzed secondary nucleation processes, as determined by chemical kinetics, providing a mechanistic basis for its amyloid assembly. This study confirms and highlights a subclass of eukaryotic PRDs prone to forming aggregates with important physiological and pathological implications.

Annexins make up a large superfamily of calcium- and phospholipid-binding proteins that regulate many membrane-related cellular events.^1^ Annexins consist of a variable N-terminal “head” domain, which confers their functional specificity, and a conserved C-terminal core comprising multiple copies of a globular “annexin repeat” that harbors calcium- and membrane-binding sites. Among the 12 human annexins, A7 and A11 contain unusually long head domains (180 and 196 residues, respectively), which are predicted to be disordered and have short peptide motifs that bind to specific intracellular protein partners.^2,3^ The highly conserved head domain of A7 (Figure S1) comprises binding sites for multiple proteins, namely, sorcin,^4^ suppressor of death domains (SODD),^2^ and apoptosis-linked gene-2 (ALG-2),^5,6^ and these interactions are suggested to regulate the biological functions of A7, including calcium homeostasis, tumor suppression, apoptosis, and plasma membrane repair. Additionally, the extreme N-terminal residues of the head domain regulate A7-mediated membrane aggregation and fusion events^7,8^ and the self-association of A7 in the presence of calcium.^2^ The head domain of A11 binds to S100A6 (calcyclin) and is involved in creating cerebral A11 inclusions in patients with amyotrophic lateral sclerosis (ALS), a fatal motor-neuron disease.^9^ We recently established that the head domain of annexin A11 phase separates into spherical condensates in vitro, which slowly transform into β-sheet-rich amyloid fibrils.^9^ Although there is little sequence similarity between the head domains of A7 and A11 (Figure S2), like A11, the A7 head domain is rich in proline residues (Figure 1A,B). Additional sequence analyses indicated that the A7 head domain may behave like other well-characterized domains that undergo phase separation and/or fibrillization (Figure S3). Thus, we investigated its aggregation properties and showed that this proline-rich domain (PRD) induces phase separation and fibrillization of A7.

**Figure 1:**
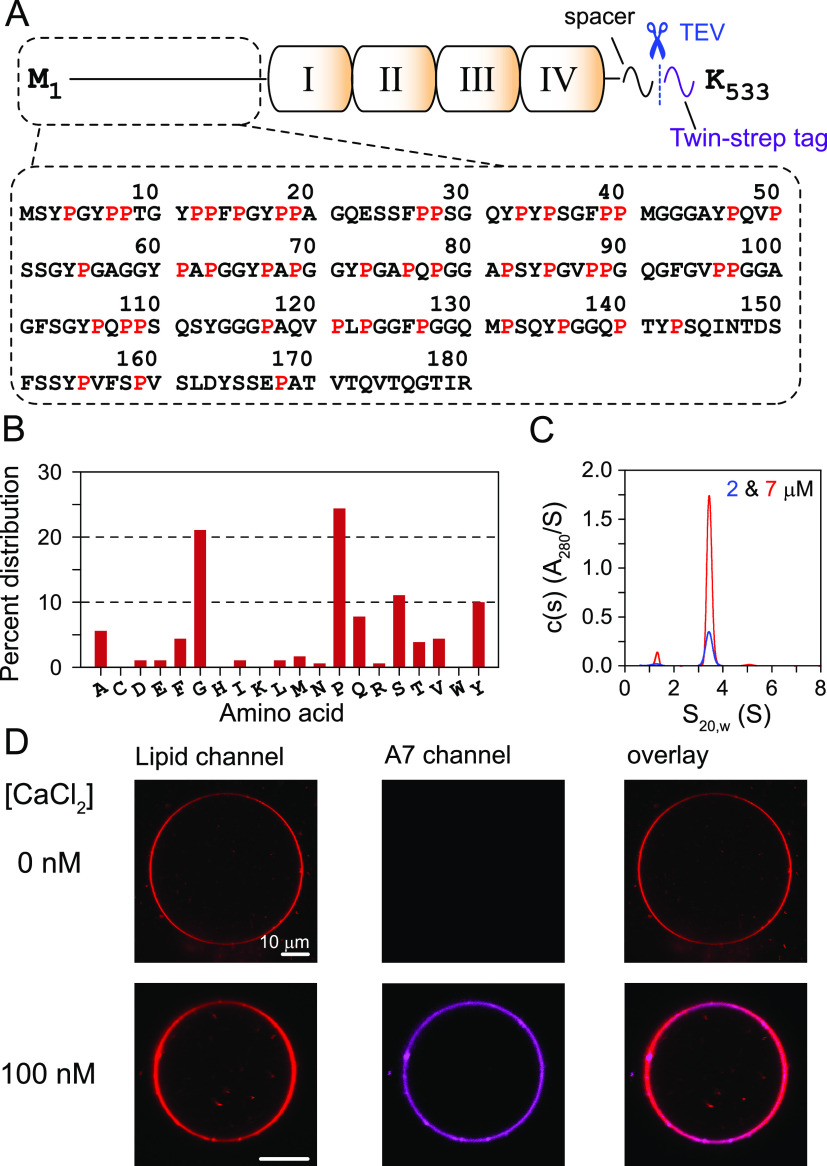
(A) Scheme of the full-length A7 construct used in this study and primary sequence of the A7 head domain: I–IV (annexin repeats), prolines (red), twin-strep tag (magenta), and TEV-protease cleavage site (dashed lines and scissors in blue). (B) Amino acid composition of the A7 head domain. (C) Sedimentation profiles of A7. (D) Fluorescence microscopy analysis of membrane-binding properties of ATTO-647N-labeled A7 (200 nM), in the absence and presence of 100 nM calcium. GUVs were made using DOPC (69.95%) and DOPS (30%) and spiked with a 16:1 Liss Rhod PE dye (0.05%). Representative microscopy images of the respective fluorescent channels and their overlays are shown. Results in panels C and D were acquired with TEV-cleaved A7.

Figure S4 lists the recombinant A7 constructs used in this study, including full-length A7 and its truncated fragments representing A7-PRD. Sedimentation velocity experiments established that, in the absence of calcium and at low concentrations (≤15 μm), recombinant A7 was predominantly monomeric (Figure 1C and Figure S5). To probe its membrane-binding properties, we monitored the colocalization of A7 with giant unilamellar vesicles (GUVs) by using confocal microscopy. ATTO-647N-labeled A7 (200 nM) was incubated with GUVs comprising zwitterionic DOPC and fluorescent Liss Rhod PE in the presence of calcium. Under these conditions, no fluorescence signal from A7 was observed at the membrane surface, establishing that A7 did not colocalize with these zwitterionic GUVs (Figure S6). Similarly, without calcium, A7 did not colocalize with GUVs comprising DOPC and negatively charged DOPS (Figure 1D). However, a robust and uniform colocalization of A7 was observed with anionic GUVs in the presence of 100 nM calcium (Figure 1D). These observations establish that recombinant A7 associates with anionic membranes in a calcium-dependent manner. These results are consistent with the original reports that showed that A7 is a calcium-dependent membrane-binding protein^10,11^ and establish that the recombinant A7 made here is functionally relevant.

At concentrations ranging between 30 and 40 μM and in the presence of calcium, A7 solutions became cloudy. Microscopy examination revealed the presence of spherical condensates (Figure 2A). A7 condensates rarely fused, suggesting a nondynamic nature. Poor fluorescence recoveries after photobleaching (FRAP) were observed for A7 condensates [∼10% average recovery in 75 s (Figure 2B)], further confirming their nondynamic character. To determine whether A7 condensates undergo a transition into amyloid fibrils, we monitored aggregation kinetics using an amyloid-sensitive dye, thioflavin T (ThT). A7 samples (*n* = 3) were incubated with calcium at 37 °C for ∼70 h, and the ThT signal was recorded with continuous linear shaking. All A7 samples exhibited sigmoidal aggregation profiles consisting of an initial dip in ThT signals, followed by lag, growth, and plateau phases, a hallmark of amyloid formation,^12^ with ∼40 h required to reach the half-maximal signal [*t*_1/2_ (Figure 2C)]. Analysis of these samples at the plateau phase by transmission electron microscopy (TEM) revealed the presence of ropelike amyloid fibrils (Figure 2C). These results establish that recombinant A7 spontaneously phase separates with calcium and undergoes a slow, time-dependent fibrillization under mechanical agitation.

**Figure 2:**
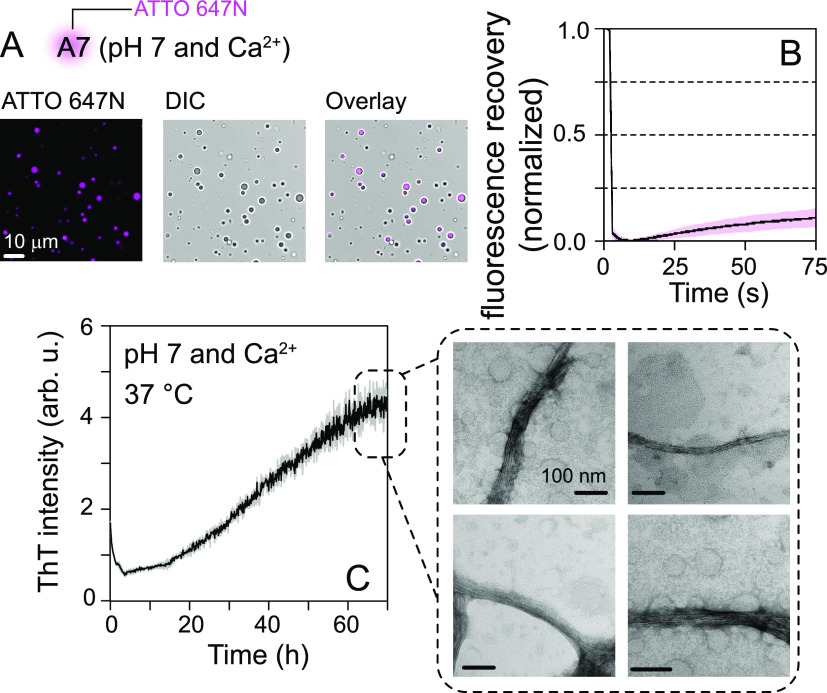
(A) Microscopy images of condensates of ATTO-647N-labeled A7 (DIC, differential interference contrast). (B) FRAP curves of A7 condensates (*n* = 8): mean (solid line) and standard deviation (SD) (shaded region). (C) Aggregation kinetics of A7 (40 μM) monitored using ThT fluorescence (*n* = 3): mean (solid line) and SD (shaded region). Representative TEM images of samples at 70 h are shown on the right (dashed square).

To determine the role of A7-PRD in the phase separation and fibrillization properties of A7, we used two truncated constructs, A7_PRD_^Strep^ and A7_PRD_ (Figure 3A). Both constructs carried a cleavable N-terminal fusion tag comprising the B1 domain of protein G (GB1), followed by a polyhistidine affinity tag and a TEV-protease cleavage site (Figure S4). Additionally, A7_PRD_^Strep^ carried a noncleavable strep affinity tag at the C-terminus, engineered to facilitate Alexa-Fluoror488 labeling for fluorescence microscopy. The latter showed that A7_PRD_^Strep^ (50 μM) phase separated in the presence of calcium (Figure 3B). Turbidity and fluorescence microscopy measurements established the ready-phase separation of A7_PRD_^Strep^ with increasing concentrations of calcium and that of A7_PRD_^Strep^ (Figure S7). Similar condensates were observed for A7_PRD_ using DIC imaging (Figure S8). Fresh condensates of A7_PRD_^Strep^ showed ∼50% average FRAP recoveries in 125 s, indicating that, unlike its full-length counterpart, A7_PRD_^Strep^ condensates were relatively more dynamic, likely because of rapid cycling between its phase-separated and soluble states and/or fluent diffusion of A7_PRD_^Strep^ molecules within the condensates (Figure 3C). The nondynamic nature of the condensates of recombinant A7 perhaps stems from other intermolecular interactions absent in its truncated counterpart. The increased level of gelation of A7 relative to its PRD is consistent with our recent observations with human protein ALIX, where we showed that condensates of ALIX were relatively more gel-like compared to those of its isolated PRD.^13^ Solutions of the A7_PRD_^Strep^ and A7_PRD_ condensates underwent visible precipitation over time. Spectral-shift assays performed using an amyloid-specific dye, Congo red (CR), showed clear red shifts for aged solutions of A7_PRD_, indicating the presence of amyloidogenic aggregates (Figure 3D).^12^ Further analyses of aged A7_PRD_ solutions by fluorescence microscopy and TEM revealed ribbon-like fibrils (panels E and F, respectively, of Figure 3). Powder X-ray diffraction of these fibrils showed two rings at 4.69 and 10.34 Å (Figure 3G), consistent with a cross-β-sheet-rich amyloid structure.^14^ To gain a mechanistic understanding of A7_PRD_ amyloid formation, we monitored its aggregation kinetics using ThT assays. With calcium and under non-agitated conditions at 37 °C, A7_PRD_ exhibited sigmoidal aggregation profiles with a *t*_1/2_ of ∼1 h (Figure 3H). Therefore, in contrast to recombinant A7 that underwent slow fibrillization under agitated conditions [*t*_1/2_ ∼ 40 h (cf. Figure 2C)], fibrillization of its isolated PRD not only was ∼40-fold faster but also did not require mechanical agitation. Although similar sigmoidal aggregation profiles were obtained in the absence of calcium for A7_PRD_, the corresponding *t*_1/2_ was notably longer, ∼24 h, establishing that calcium accelerates the aggregation of A7_PRD_. Collectively, these results show that A7-PRD forms spherical condensates that rapidly mature into β-sheet-rich fibrils and are likely responsible for phase separation and fibrillization of full-length A7.

**Figure 3:**
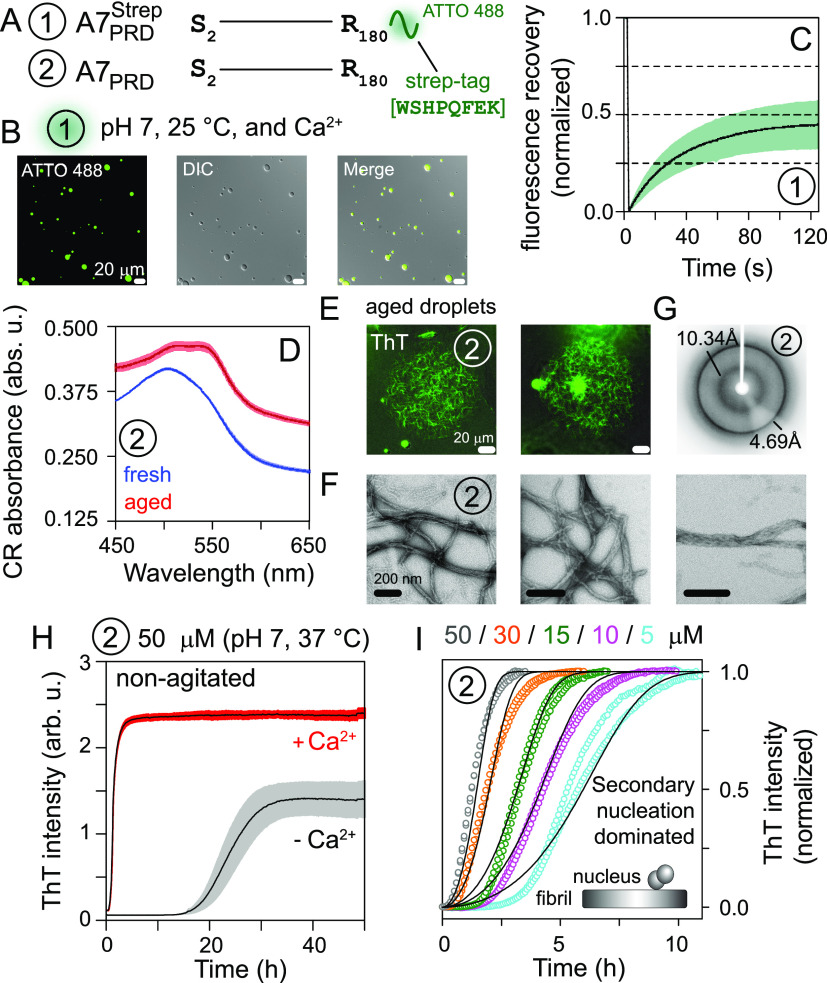
(A) A7-PRD constructs tested in this study. Each construct is designated by a circled number. (B) Microscopy images of condensates of ATTO-488-labeled A7_PRD_^Strep^. The circled number signifies the construct shown in panel A. (C) FRAP curves of A7_PRD_^Strep^ condensates (*n* = 8): mean (solid line) and SD (shaded region). (D) CR absorbance spectra of A7_PRD_ aggregates (red) and freshly prepared solutions (blue) (*n* = 3). (E) Fluorescence microscopy, (F) TEM, and (G) X-ray diffraction of A7_PRD_ fibrils. (H) Aggregation kinetics of A7_PRD_, in the absence (gray) and presence (red) of calcium, monitored by a ThT assay (*n* = 3): mean (solid line) and SD (shaded region). (I) Aggregation kinetics of A7_PRD_ at different concentrations (*n* = 2): experimental data (circles) and global fits carried out using model “secondary nucleation dominated, unseeded” in the program Amylofit (solid lines).^15^ The inset shows a scheme of the secondary nucleation process in which a nucleus comprising two monomers (gray circles) adds to the existing fibril (gray rectangle).

To elucidate the underlying microscopic processes that contribute to the fibrillization of A7_PRD_, we carried out ThT assays by varying the initial monomer concentrations. Highly reproducible curves with minimal variation between the replicates (*n* = 2) were obtained at each concentration point, namely, 5, 10, 15, 30, and 50 μM (Figure S9). The high quality of these data sets facilitated their global fitting using the protocol described by Knowles and colleagues via the online platform AmyloFit.^15^ Briefly, first, the relationship between *t*_1/2_ and the starting monomer concentration of A7_PRD_ (*m*_0_) was ascertained by fitting the power-law function *t*_1/2_ ∼ *m*_0_^γ^, where γ is the scaling exponent (Figure S10). The corresponding γ value was −0.66, suggesting that A7_PRD_ aggregation proceeds by a secondary process with a weak monomer concentration dependence, such as fibril-catalyzed secondary nucleation or fragmentation.^16^ Next, data fitting was performed, where different kinetic models were fit globally to the experimental ThT curves. The model termed “secondary nucleation dominated, unseeded”, which incorporates primary and secondary nucleation along with fibril elongation, adequately replicated the experimental ThT curves. Note that simpler models that did not incorporate secondary nucleation (Figure 3I; also see Figure S11 for a schematic of individual processes involved in fibrillization) did not fit well. The secondary nucleation model yielded two effective rates, λ and κ, where λ denotes the rate of fibril formation via primary nucleation and elongation while κ describes the rate of fibril formation via elongation and secondary nucleation. The comparison of these two rates as a function of A7_PRD_ concentration is shown in Figure S12. The values of λ as a function of A7_PRD_ concentration, ranging from 0.1 to 0.7 h^–1^, were consistently lower than those of κ, which ranged from 0.5 to 1.8 h^–1^, demonstrating that fibrillization of A7_PRD_ involves both primary and secondary nucleation, with the latter being the dominant process. The addition of low concentrations of preformed fibrils (i.e., seeds; 1% and 5%) reduced the lag phase of aggregation of 5 μM A7_PRD_ by ∼4-fold (Figure S13), further confirming that A7_PRD_ aggregation is dominated by secondary nucleation.^17^

In summary, we show that the A7_PRD_ phase separates and forms amyloid fibrils in vitro. It is, thus, likely responsible for the phase separation and fibrillization of full-length A7 observed here. A7 is known to self-associate and promote membrane aggregation and fusion events with calcium.^18−20^ Prior studies have ascribed these properties to the extreme N-terminal residues of A7_PRD_.^2,7,8^ The results presented here are consistent with these observations. The aggregation properties of A7_PRD_ are like those of A11_PRD_ despite their limited sequence similarity. We found that the amyloid fibrils formed by A11_PRD_ were labile and slowly dissolved upon the addition of its binding partner, S100A6.^9^ Studies to determine whether A7_PRD_ fibrils exhibit a similar dissolution pattern with its binding partners, namely, sorcin, SODD, and ALG-2, are currently ongoing in our laboratory. Finally, we note that the aggregation properties of A7_PRD_ are similar to those of another PRD discovered in our laboratory, belonging to the human protein ALIX, which phase separates and forms amyloid fibrils.^13,21−23^ Therefore, although eukaryotic PRDs are known to create dynamic cell signaling networks, PRDs of A7, A11, and ALIX may represent a new subclass of PRDs capable of phase separation and fibrillization.

## Supporting Information Available

Experimental details (Materials and Methods); primary sequence comparison of different A7 head domains and head domains of A7 and A11, comparison of A7_PRD_ with other aggregation-prone proteins, recombinant A7 constructs, analyses of their properties by sedimentation and turbidity assays and fluoroscence microscopy and spectroscopy, a scheme of processes involved in fibrillization, comparison of the rates of the primary and secondary processes of A7_PRD_ fibrillization, and seeding experiments (Figures S1–S13); and recombinant A7 constructs used in this study (Table S1) (PDF)

## Accession Codes

Human annexin A7 (UniProt entry P20073). All A7 plasmids reported in this study were deposited in the Addgene repository (https://www.addgene.org) [accession nos. 198635 (A7), 190066 (A7_PRD_), and 190067 (A7_PRD_^Strep^)].
