# Supplementary material for: Phase Separation and Fibrillization of Human Annexin A7 Are Mediated by Its Proline-Rich Domain
Source: Biochemistry. 2023 Oct 3;62(21):3036–40. doi: 10.1021/acs.biochem.3c00349 (PMC10634317; doi:10.1021/acs.biochem.3c00349)
Supplement: Supplementary file 1 — bi3c00349_si_001.pdf [file bi3c00349_si_001.pdf]

Supporting Information

Phase separation and fibrillization of human annexin A7 are mediated by its proline-rich domain.

Chenrong Yu,[1§] Spencer L. Nelson,[1§] Georg Meisl,[2] Rodolfo Ghirlando,[3] and Lalit Deshmukh[1,*]

[1]Department of Chemistry and Biochemistry, University of California San Diego, La Jolla, CA 92093, USA, [2]Department of Chemistry, University of Cambridge, Cambridge CB2 1EW, UK, [3]Laboratory of Molecular Biology, National Institute of Diabetes and Digestive and Kidney Diseases, National Institutes of Health, Bethesda, MD 20892, USA.

[§]Equal contribution.

[*]To whom correspondence should be addressed. Email: ldeshmukh@ucsd.edu

This PDF file includes:

Materials and Methods

Supplementary Figures S1 to S13

Supplementary Table S1

Supplementary References (1 to 23)

Materials and Methods

Materials.

ATTO-647N maleimide was purchased from ATTO-TEC GmbH (catalog no. AD 647N-41) and was dissolved in dimethylformamide (DMF) at a concentration of ~11.5 mM. Streptavidin Alexa-Fluor488 conjugate was purchased from Thermo Fisher Scientific (catalog no. S32354). mPEG20K-Silane and Congo red (CR) were purchased from Sigma-Aldrich (catalog no. JKA3100 and C6277, respectively). Thioflavin T (ThT) and gels for sodium dodecyl sulfate polyacrylamide gel electrophoresis (SDS-PAGE; 4–12% Bis-Tris gels) were purchased from Thermo Fisher Scientific (catalog no. AC211760050, and NW04122BOX, respectively). Reagents for the preparation of giant unilamellar vesicles (GUVs), namely 1,2-dioleoyl-sn-glycero-3-phosphocholine (DOPC), 1,2-dioleoyl-sn-glycero-3-phospho-L-serine (DOPS), and 1,2-dipalmitoyl-sn-glycero-3-phosphoethanolamine-N-(lissamine rhodamine B sulfonyl) [i.e., 16:0 Liss Rhod PE], were purchased from Avanti polar lipids (catalog no. 850375, 840035, and 810158, respectively).

Methods.

Recombinant protein expression and purification.

Codon-optimized A7 constructs, namely A7, $A7_{PRD}$, and $A7_{PRD}^{Strep}$, were custom synthesized from Azenta Life Sciences; see Fig. S4A for the design and mass-spectrometry (MS) analysis and Table S1 for subcloning of each construct. A7 was expressed with a tobacco etch virus (TEV) cleavable C-terminal twin-strep tag.[1] The primary sequence of the twin-strep tag is as follows: GSGSGSGSA<u>WSHPQFEK</u>GGGSGGGSGGSA<u>WSHPQFEK</u>; underlined residues represent the binding motif for strep-tactin, a derivative of streptavidin. Both truncated constructs, namely $A7_{PRD}$, and $A7_{PRD}^{Strep}$, were expressed with the N-terminal B1 domain of protein G (GB1) tag,[2] used to enhance the expression levels, followed by a spacer sequence, a polyhistidine (6xHis) affinity tag, and a TEV cleavage site. Additionally, $A7_{PRD}^{Strep}$ carried a non-cleavable C-terminal strep tag.

The primary sequence of the strep tag is as follows: WSHPQFEK. All A7 plasmids reported in this study were deposited in the Addgene repository, https://www.addgene.org (accession no. 198635 [A7], 190066 [$A7_{PRD}$], and 190067 [$A7_{PRD}^{Strep}$]). The construct for TEV protease was a generous gift from David S. Waugh (NIH).

TEV protease was expressed at 37 °C as described previously.[3] All A7 constructs were expressed at 16 °C. Cells were grown at 37 °C in 1 L Luria-Bertani (LB; MP Biomedicals, catalog no. 3002-036) medium at natural isotopic abundance. About 30 min before induction, the temperature of the cell culture was reduced to 16 °C. Cells were induced with 1 mM isopropyl β-d-1-thiogalactopyranoside (IPTG) at an absorbance of 0.8 at 600 nm and harvested after ca. 24 h.

The purification scheme of TEV protease has been described previously.[3] A7 was purified using affinity chromatography (ÄKTA Pure protein purification system, Cytiva) whereas $A7_{PRD}$, and $A7_{PRD}^{Strep}$, were purified using a combination of affinity and size-exclusion chromatography (ÄKTA Pure and Start protein purification systems, Cytiva) and reverse-phase high-performance liquid chromatography (HPLC; 1260 Infinity II liquid chromatography system, Agilent Technologies). In the case of A7, cells were resuspended in a lysis buffer comprising 50 mM Tris, pH 8, 10 mM ethylenediaminetetraacetic acid (EDTA), and 5 mM β-mercaptoethanol (BME). Cells were lysed using an EmulsiFlex-C3 (Avestin) and cleared by centrifugation (48,380g, 30 min). The resultant supernatant was loaded onto a XK 16/20 chromatography column (Cytiva) prepacked with Strep-Tactin-XT Sepharose resin (Cytiva) pre-equilibrated with 50 mM Tris, pH 8.0, 10 mM EDTA, and 5 mM BME, and eluted in the same buffer containing 50 mM biotin. The eluted protein was mixed with recombinant TEV protease (molar ratio 50:1) to hydrolyze the C-terminal twin-strep tag. The reaction mixture was dialyzed overnight at 4 °C in a buffer comprising 25 mM (N-2-hydroxyethylpiperazine-N'-2-ethanesulfonic acid) [HEPES], and 1 mM (tris(2-carboxyethyl)phosphine) [TCEP]; Slide-A-Lyzer G2 dialysis cassettes (Thermo Fisher Scientific). The completion of proteolysis was assessed using SDS-PAGE electrophoresis. The hydrolyzed product was passed through Strep-Tactin-XT and HisTrap (Cytiva) columns, pre-equilibrated with

the above-mentioned buffer used for dialysis. Relevant flow-through fractions were pooled, concentrated, and stored at -80 °C.

For $A7_{PRD}^{Strep}$, cells were resuspended in a lysis buffer comprising 50 mM Tris, pH 8, and 6 M guanidine hydrochloride (GdmCl). Cells were lysed by EmulsiFlex-C3 and cleared by centrifugation. The resultant supernatant was filtered through a 0.45 µm vacuum-driven filtration device (Stericup, Sigma-Aldrich) before being loaded onto a HisTrap column (Cytiva) pre-equilibrated with lysis buffer. Bound protein was washed with 10 column volumes of refolding buffer comprising 50 mM Tris, pH 8.0, and 250 mM NaCl and eluted in the same buffer containing 1 M imidazole. The eluted protein fractions were pooled and loaded onto a XK 16/20 chromatography column (Cytiva) prepacked with Strep-Tactin Sepharose resin (Cytiva) pre-equilibrated with 50 mM Tris, pH 8.0, and 250 mM NaCl, and eluted in the same buffer containing 2.5 mM d-desthiobiotin. The eluted protein was mixed with recombinant TEV protease (molar ratio 50:1) to hydrolyze the N-terminal GB1 fusion tag (completion of proteolysis was assessed using SDS-PAGE electrophoresis). The proteolysis reaction was carried out at room temperature (~20 h) and produced a poorly soluble hydrolyzed product. The precipitated product was solubilized by the addition of 6 M GdmCl and further purified using reverse-phase HPLC (Jupiter 10 µm C18 300 Å column) with a 25–42% acetonitrile gradient comprising 0.1% trifluoroacetic acid (TFA). The eluted protein fractions were pooled, lyophilized, and stored at -80 °C.

For $A7_{PRD}$, a similar lysis and HisTrap column purification procedures as described above for $A7_{PRD}^{Strep}$ were carried out. The eluted protein was further purified using size-exclusion chromatography on a HiLoad 26/600 Superdex 75 prep-grade column (Cytiva) pre-equilibrated with 50 mM Tris, pH 8.0, and 250 mM NaCl. Relevant $A7_{PRD}$ fractions were pooled and incubated with recombinant TEV protease to cleave off the N-terminal GB1 fusion tag. The hydrolyzed product was further purified by reverse-phase HPLC using the above-described conditions. Eluted $A7_{PRD}$ fractions were aliquoted, lyophilized, and stored at -80 °C.

All protein constructs were verified by mass-spectrometry (MS) as described before.[3-5]

Sedimentation velocity analytical ultracentrifugation (AUC).

Sedimentation velocity experiments on A7 were carried out at 50,000 rpm and 20 °C on a Beckman Coulter ProteomeLab XL-I analytical ultracentrifuge and an An-50-Ti rotor following standard protocols.[6] A7 stock solution was prepared as described above (see Recombinant protein expression and purification section). Samples were diluted to ~2 and ~7 μM using a buffer comprising 25 mM HEPES, pH 7, and 1 mM TCEP. Absorbance sedimentation data were collected at 280 nm and analyzed using our published protocols.[3, 7-9] Sedimentation profiles showed the presence of a monomeric A7.

Fluorophore labeling, phase separation, and turbidity assays.

In the case of A7, protein was mixed with a 3-molar equivalent of ATTO-647N maleimide in 25 mM HEPES, pH 7, and 1 mM TCEP. The reaction was allowed to proceed at 4 °C overnight. The excess dye was removed by PD midiTrap G-25 columns (Cytiva). This sample was mixed with the corresponding unconjugated protein (concentration of fluorophore-labeled protein = 5 molar percent). 2 mM $CaCl_2$ was then added to induce phase separation of A7 and the resultant sample (final protein concentration = 40 μM) was visualized using fluorescence microscopy (see below).

For $A7_{PRD}^{Strep}$, the lyophilized protein was dissolved in a small volume of dimethylsulfoxide (DMSO) and diluted immediately in a buffer containing 25 mM HEPES and 5 mM $CaCl_2$ to induce phase separation (final protein concentration = 50 μM). 0.02 mg/mL Streptavidin Alexa-Fluor488 conjugate was then added to this solution, which was visualized using fluorescence microscopy (see below). To determine the effects of calcium and protein concentration on the phase separation of $A7_{PRD}^{Strep}$, the turbidity of $A7_{PRD}^{Strep}$ solutions with varying protein and calcium concentrations was recorded at an optical density (OD) of 330 nm using a DU 730 UV-Vis spectrophotometer (Beckman Coulter) in 1 cm quartz cuvettes (Starna Cells Inc.). Additionally, these samples were visualized using fluorescence microscopy. The condensates of $A7_{PRD}$ formed in the presence of calcium were monitored using differential interference contrast (DIC) imaging. $A7_{PRD}$ condensates were prepared using the same procedure as that of $A7_{PRD}^{Strep}$, sans Streptavidin Alexa-Fluor488.

Microscopy imaging and FRAP assays.

Microscopy imaging and FRAP assays were carried out using our previously published protocols.[10-12] Briefly, DIC imaging was performed on a Nikon Ti2 widefield microscope equipped with a DS-Qi2 CMOS camera and 100x/1.49NA oil DIC N2 Objective; Nikon Imaging Center, UC San Diego. The condenser prism and the polarizer cube were controlled by the Nikon Elements software. Samples of A7 condensates were excited by a 640 nm laser controlled by a Lumencor SpectraX for imaging of ATTO-647N. In the case of $A7_{PRD}^{Strep}$ + Streptavidin Alexa-Fluor488 mixtures, a 488 nm laser was used. Microscopy image of aged droplets of $A7_{PRD}$ shown in Fig. 3E, main text, was acquired using a similar procedure. Briefly, droplets of 50 µM $A7_{PRD}$ with 20 µM ThT were incubated at 37 ºC for one day. Images were taken using a 488 nm laser.

For microscopy experiments, slides were passivated using PEG-silane.[12-13] Briefly, slides were cleaned by sonication in 3% v/v Hellmanex III (Sigma-Aldrich), rinsed in 0.5 M NaOH, dried using a nitrogen stream, and placed in a vacuum oven (VWR) at 90 °C for 10 min. PEG-Silane was dissolved in DMSO at 5% w/v and was sandwiched between the slide and coverslip, and incubated at 90 °C for 20 min. Passivated slides were subsequently rinsed with water and dried with a nitrogen stream. Imaging chambers composed of two passivated coverslips and a 9-mm silicone mold (Grace BioLabs) were used for all microscopy analyses.

FRAP measurements of ATTO-647N-labeled A7 were performed on a Nikon point scanning confocal C2 with 2 GaAsP PMTs using a Plan Apo λ 100x/1.45 NA Oil objective. Photobleaching of each sample was achieved using 2 iterations of 50% 640 nm laser power directed at the bleaching area for 10 s, and subsequent recovery was imaged at 2 s intervals over 150 frames using 0.1% 640 nm laser power. Images were corrected for background fluorescence, and intensity from the bleached region was normalized against an unbleached region on a nearby condensate of similar size and intensity. A similar procedure was used for $A7_{PRD}^{Strep}$ + Streptavidin Alexa-Fluor488 mixtures, except for the use of 488 nm laser.

GUV preparation.

GUVs were prepared using the inverted emulsion method.[14] Briefly, for the oil phase, the chloroform solutions of DOPC, DOPS, and 16:0 Liss Rhod PE were mixed in a glass vial under dark conditions (69.5, 30, and 0.5%, respectively). The resultant mixture was dried under nitrogen, followed by vacuum desiccation (vacuum oven; VWR) for ~2 h at room temperature. 1 mL of mineral oil (Thermo Fisher Scientific, catalog no. O121-1) was added to the vial and sonicated for 1 h at room temperature. For the aqueous phase, phosphate buffered saline (PBS; osmolarity: 280 mOsm, pH 7.4) was mixed with Ficoll 400 (Sigma-Aldrich; 35% w/v in water) to give a final concentration of 3.5% Ficoll-400. 10 μL of this solution was added to 100 μL oil phase and emulsified. The emulsion was then mixed with the aqueous phase, followed by centrifugation (10,000g, 10 min, 4 °C). The GUVs at the bottom of the tube were collected and stored at 4 °C for further analysis.

CR assay.

CR assay was carried out as described previously.[4-5, 12] Briefly, CR was dissolved in MilliQ water (MilliQ IQ 7000 purification system, Millipore–Sigma). CR stock solution (0.2% w/v) was filtered through a 0.22 μm filter and used immediately. $A7_{PRD}$ samples were prepared by dissolving ~1 mg lyophilized protein in 20 μL DMSO and diluting this solution to 50 μM using a buffer comprising 25 mM HEPES, pH 7.0, and 5 mM $CaCl_2$. These freshly prepared samples were mixed with CR stock solution (50:1 dilution; protein vs. CR). Additionally, samples of $A7_{PRD}$ were incubated for ~3 h at room temperature to induce fibrillization, followed by mixing with CR stock solution (same dilution as above). In both cases, the absorption spectra of three replicates were measured using an DeNovix DS-11+ (M/C) Spectrophotometer.

Transmission electron microscopy (TEM).

TEM samples of fibrils of A7 and $A7_{PRD}$ constructs were prepared using our published protocols.[4-5] TEM images were acquired using a JEM-1400 Plus transmission electron microscope (JEOL) and recorded on a OneView digital camera (Gatan), Electron Microscopy Core Facility, UC San Diego.

X-ray diffraction.

X-ray diffraction was carried out as described previously.[12] A 50 μM stock solution of A7$_{PRD}$ was incubated at 37 °C for a week. A7$_{PRD}$ fibrils were pelleted for 30 min at 259,000g and 20°C using Optima XE Ultracentrifuge and SW 55 Ti swinging bucket rotor (Beckman Coulter). A small amount of the sample was dried and loaded onto a Cryoloop. The sample was mounted on a Bruker Microstar 592 diffractometer equipped with an APEX II CCD detector and Cu Kα radiation (λ = 1.54178 Å); UC San Diego Crystallography Facility. X-ray data was collected using a 360° ϕ scan with an exposure time of 300 s.

Fibril formation and dissolution kinetics.

In the case of A7$_{PRD,}$ samples were prepared by dissolving the lyophilized A7$_{PRD}$ in DMSO and rapidly diluting the DMSO stock in a buffer comprising 25 mM HEPES, pH 7, and 5 mM $CaCl_2$ to achieve final concentrations ranging from 5–50 μM. Measurements were carried out at 37 °C under non-agitated conditions using a microplate reader (Infinite M Plex; Tecan) and sealed 96-well flat bottom plates (Corning; catalog no. 3370) containing 100 μl sample per well. ThT (20 μM) fluorescence was recorded as a function of time. Excitation and emission wavelengths were 415 and 480 nm, respectively. For A7, similar experimental conditions were used, except that measurements were carried out with continuous linear shaking (1.5 mm, 335.8 rpm). To determine the effect of seeding on the aggregation kinetics, 50 μM of A7$_{PRD}$ was allowed to aggregate overnight at room temperature. The resultant fibrils were resuspended using a pipette tip and were added to the freshly prepared 5 μM A7$_{PRD}$ solutions, such that the seeds represented 1 and 5% of the total protein mass. These samples were allowed to aggregate under non-agitating conditions; 5 μM A7$_{PRD}$ solution without the seeds served as a control. Experimental and buffer conditions were the same as described above. Global fitting of the experimental ThT curves for A7$_{PRD}$ was carried out using the protocol described by Meisl et al.[15]

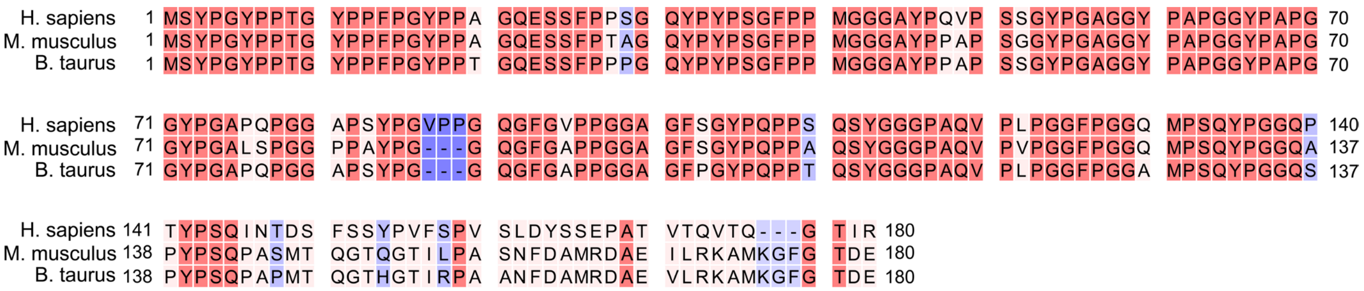

Figure S1. Primary sequence comparison of representative A7-head domains among vertebrate species. A blue-to-red gradient is used to denote conservation, with blue and red colors depicting the least and most conserved residues, respectively. The following sequences were used for analysis: H. sapiens (Uniprot accession no. P20073), M. musculus (Uniprot accession no. Q07076), and B. taurus (Uniprot accession no. P20072).

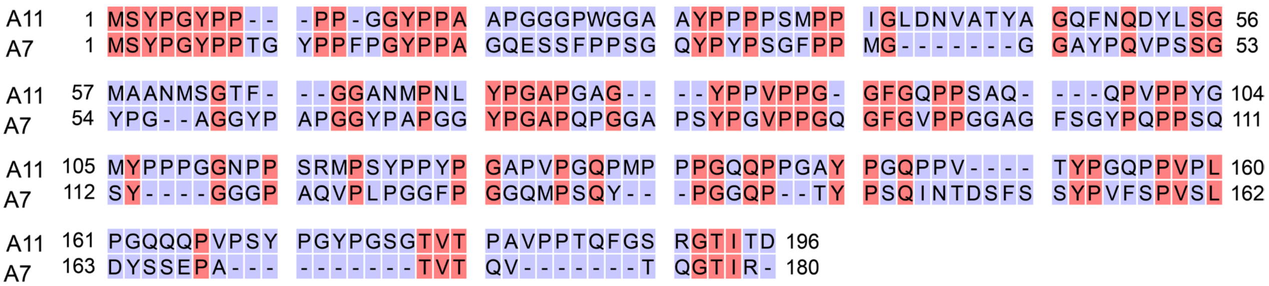

Figure S2. Primary sequence comparison of head domains of A7 and A11. Same color gradient as Fig. S1. The uniprot accession no. for A11 is P50995.

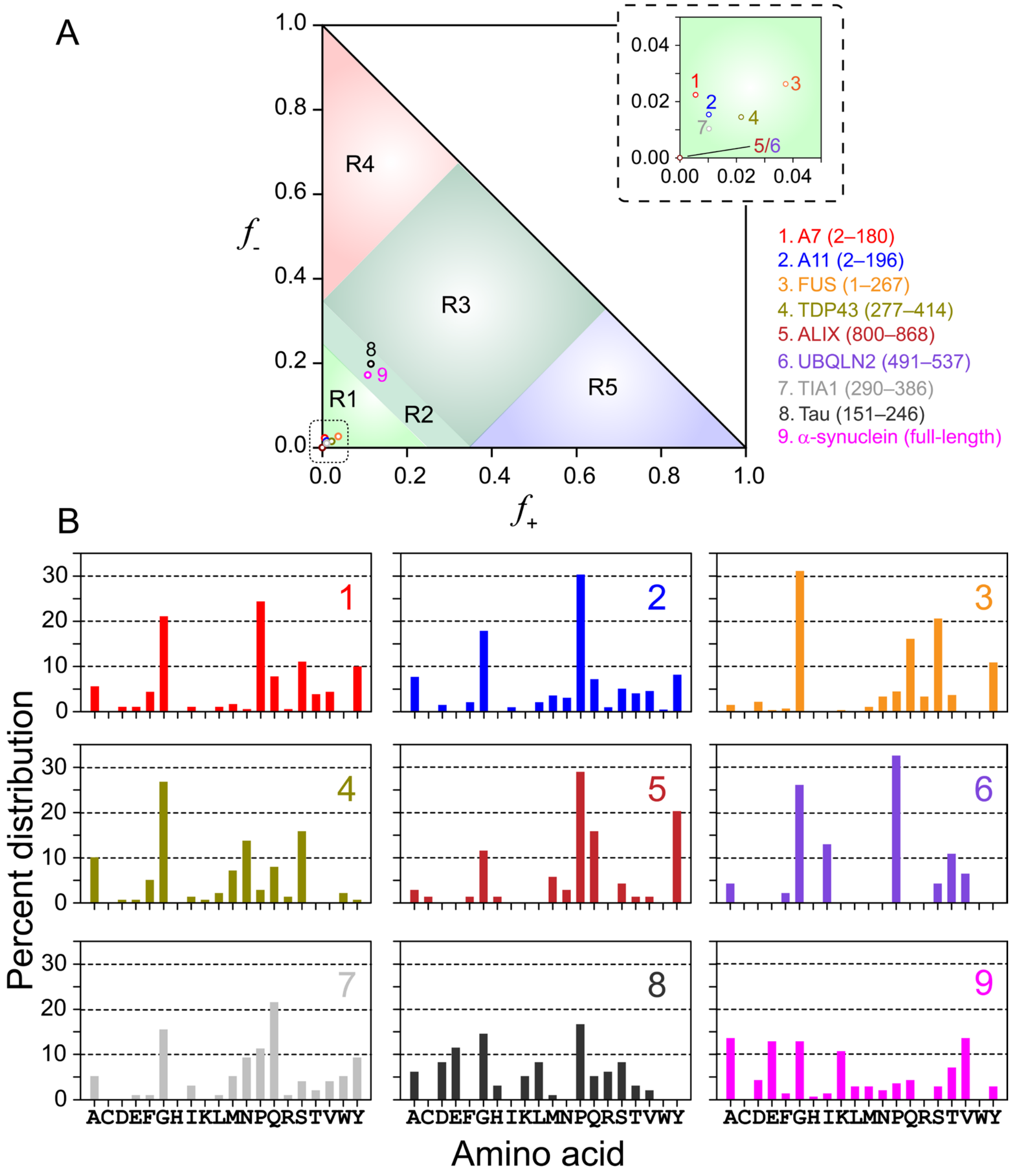

Figure S3. Comparison of A7-head domain with other known human protein domains/regions that undergo phase separation and/or fibrillization. (**A**) Analysis of the primary sequence of A7-head domain using the webserver CIDER (classification of intrinsically disordered ensemble relationships)[16] and the corresponding diagram of states. The CIDER webserver calculates the fractions of positively and negatively charged residues in a given polypeptide sequence (f_+ and f_-, respectively), and uses these fractions to partition the sequence into one of the five distinct conformational classes, namely globules and tadpoles (R1), collapsed or expanded (R2), coils and hairpins (R3), and swollen coils (R4 and R5). The A7-head domain is

compared with domains/regions of eight other proteins that drive their phase separation and/or fibrillization[4-5, 10, 12, 17-21], depicted with circles, and marked with numbers. The names of the proteins are on the right with residue numbers of the domains in parenthesis; annexin A7 (uniprot entry: P20073), annexin A11 (uniprot entry: P50995), fused in sarcoma (FUS; uniprot entry: P35637), TAR DNA-binding protein 43 (TDP43; uniprot entry: Q13148), ALG-2-interacting protein X (ALIX; uniprot entry: Q8WUM4), ubiquilin-2 (UBQLN2; uniprot entry: Q9UHD9), T-cell restriction intracellular antigen 1 (TIA1; uniprot entry: P31483), tau (uniprot entry: Q9UHD9), and α-synuclein (uniprot entry: P37840). Except for tau and α-synuclein, the remaining proteins are closely clustered, highlighted with a dashed square; an expanded image of the dashed region is shown on right. (**B**) Amino acid composition of proteins shown in panel **A**, designated with the same numbering scheme. Notable among these is the uniform abundance of glycine residues (ranging between 12–33%). In addition, the following domains/regions harbor high proline content (>15%): A7 (residues 2–180; ~24% prolines), A11 (residues 2–196; ~30% prolines), ALIX (residues 800–868; ~29% prolines), UBQLN2 (residues 491–537, ~33% prolines), and tau (residues 151–246; ~17% prolines). Note that although the precise molecular code that governs biomolecular phase separation is unclear, it is primarily mediated by disordered proteins or domains.[22] PRDs are therefore often involved in phase separation due to their lack of structure and their ability to form dynamic multivalent complexes.[10] Additionally, the presence of interspersed glycine and aromatic residues (e.g., tyrosine and phenyl alanine) may modulate phase separation of the PRDs as well as their fibrillization via increased degrees of freedom and hydrophobic CH/π interactions, respectively.

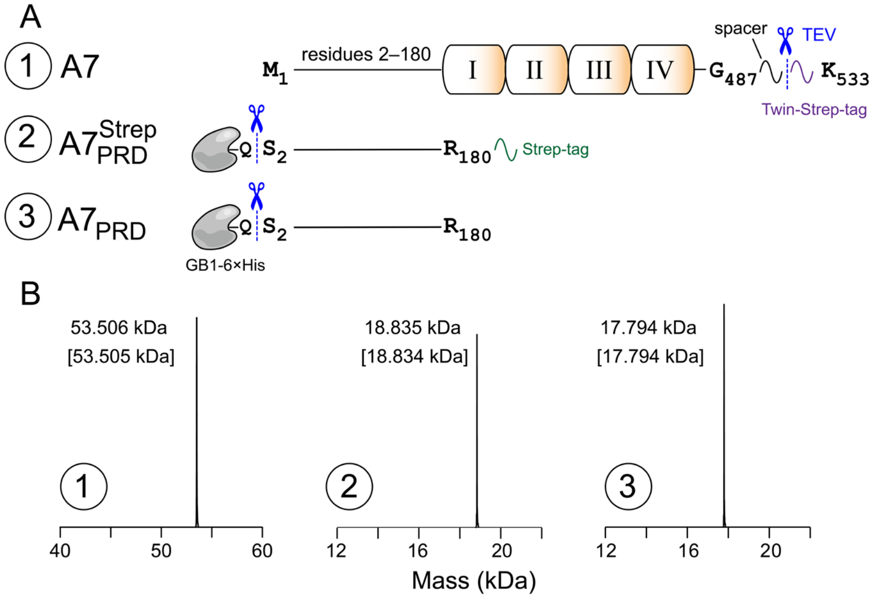

Figure S4. Recombinant A7 constructs used in current study. (**A**) List of recombinant A7 constructs, namely A7, $A7^{Strep}_{PRD}$ and $A7_{PRD}$. Each construct is designated by a circled number. The locations of the TEV cleavage sites are marked by blue vertical dashed lines and scissors. In the case of A7 (construct no. 1), the TEV cleavage site is located at the C-terminus, sandwiched between a spacer sequence (SGSENLYFQ) and the twin-strep tag[1]. Note that native residue Q488 of A7 was not included in this construct to accommodate the spacer and TEV cleavage site. For the truncated constructs, GB1-6xHis denotes the N-terminal GB1 tag, used to enhance the expression levels, followed by a spacer sequence and a polyhistidine (6xHis) affinity tag. Additionally, $A7^{Strep}_{PRD}$ (construct no. 2) carried a non-cleavable C-terminal strep tag[23]. (**B**) Analysis of TEV-cleaved constructs (same numbering as panel **A**) using liquid chromatography–electrospray ionization–time-of-flight mass spectrometry (LC–ESI–TOFMS); the numbers in parenthesis represent the corresponding theoretical masses.

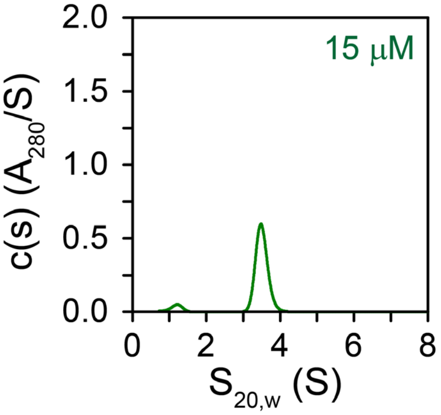

Figure S5. Sedimentation analysis of recombinant A7. Absorbance sedimentation c(s) profiles of recombinant A7 (15 μM). All measurements were carried out at 20 ºC in 25 mM HEPES, pH 7, and 1 mM TCEP. Note that unlike the profiles shown in Fig. 1C (main text) that were acquired using 12 mm cell, the above data were acquired using 3 mm cell.

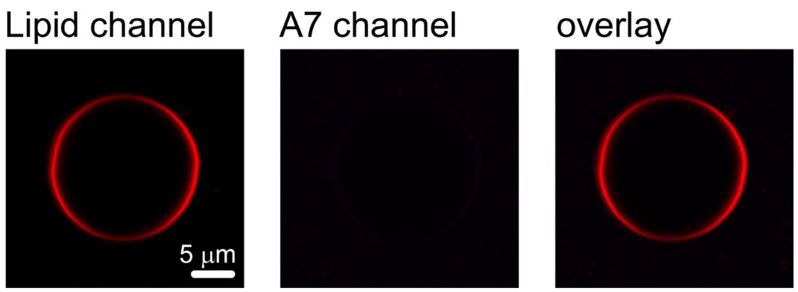

Figure S6. Confocal microscopy analysis of membrane binding properties of A7. GUVs were made using DOPC (99.95%) and spiked with 16:1 Liss Rhod PE dye (0.05%). The concentration of ATTO-647N-labeled A7 was 200 nM. The buffer conditions were as follows: 25 mM HEPES, pH 7, and 100 nM calcium. Representative microscopy images of the respective fluorescent channels and their overlay are shown. The lack of fluorescence signal from A7 at the membrane surface indicated that A7 did not colocalize with zwitterionic GUVs containing DOPC.

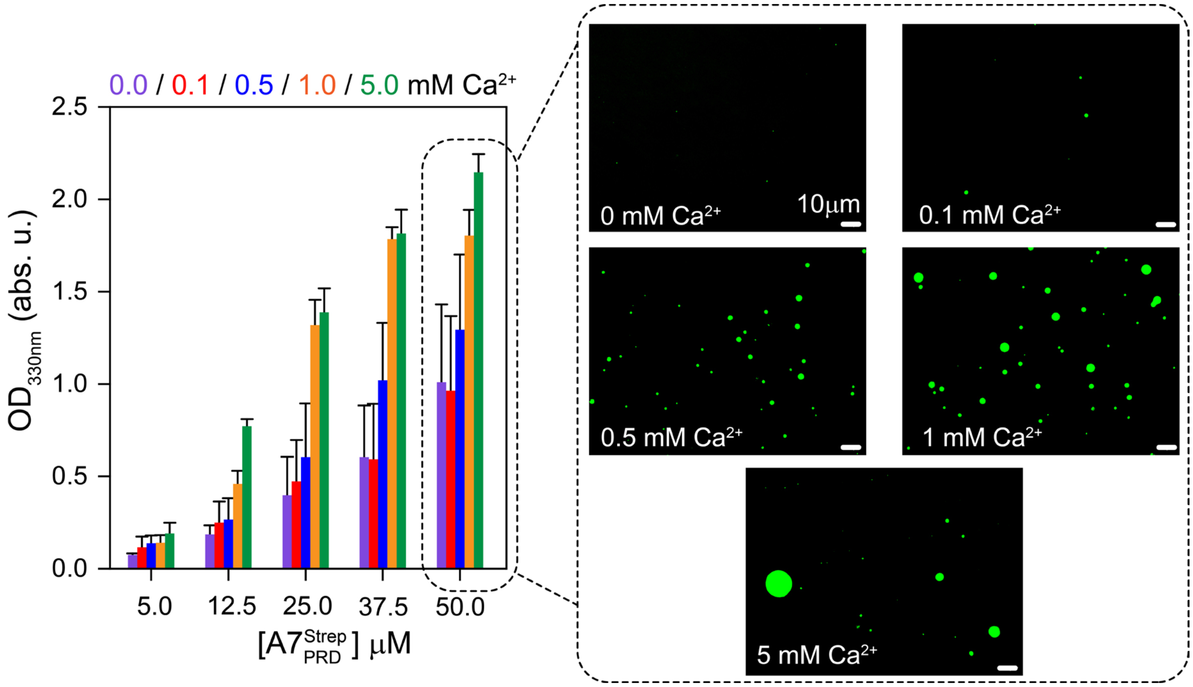

Figure S7. The interplay between calcium, protein concentration, and phase separation of $\mathbf{A7_{PRD}^{Strep}}$. Phase separation of $A7_{PRD}^{Strep}$ was monitored by turbidity assay at varying protein and calcium concentrations; $n = 2$. Representative images of 50 µM $A7_{PRD}^{Strep}$ with and without calcium are shown in the dashed square on the right. Note that in the absence and presence of 0.1 mM calcium, the condensates formed by 50 µM $A7_{PRD}^{Strep}$ were on a submicrometer scale and, thus, were difficult to visualize using fluorescence microscopy. A stepwise increase in calcium concentrations (0.5, 1, and 5 mM) resulted in the formation of correspondingly larger droplets, further confirming the regulatory roles of calcium and consequently, of hydrophobic interactions in the modulation of A7 phase separation. Microscopy images were acquired at 25 °C. The buffer composition was as follows: 25 mM HEPES, pH 7, and 0.02 mg/mL Streptavidin Alexa-Fluor488.

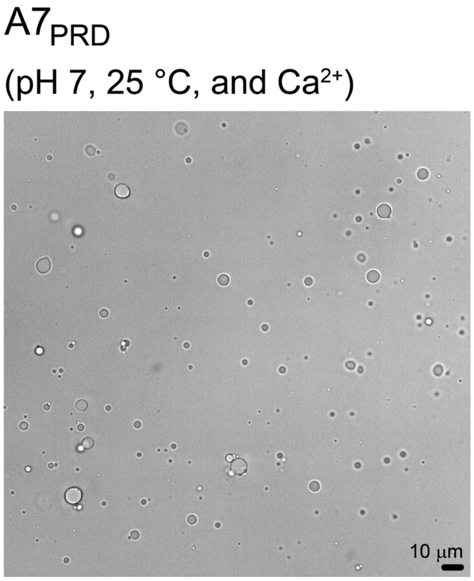

Figure S8. DIC images of droplets made by A7$_{PRD}$. Images were acquired at 25 °C. The buffer composition as follows: 25 mM HEPES, pH 7, and 5 mM calcium.

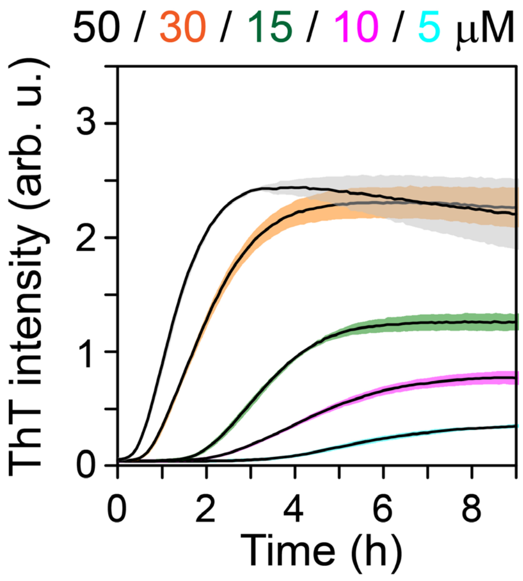

Figure S9. Aggregation kinetics of A7$_{PRD}$. ThT fluorescence was monitored to determine the effects of concentration on the aggregation kinetics of A7$_{PRD}$, $n = 2$, mean (solid line), SD (shaded region). All measurements were carried out at 37 °C under non-agitated conditions. The buffer composition as follows: 25 mM HEPES, pH 7, 5 mM calcium, and 20 μM ThT.

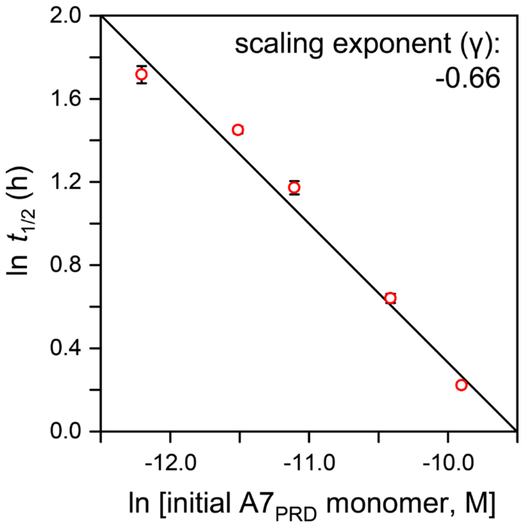

Figure S10. Dependence of half-times, $t_{1/2}$, on the initial monomer concentration of $A7_{PRD}$. Double logarithmic plot, log $t_{1/2} = \gamma \log[m_0]$ + constant, where γ is the scaling exponent and m_0 is $A7_{PRD}$ monomer at time 0. The raw data used to generate this plot is shown in Fig. S9. The values of $t_{1/2}$ and γ were extracted using the program Amylofit[15] by fitting the power-law function, $t_{1/2} \sim [m_0]^{\gamma}$. The obtained value of γ (i.e., -0.66) suggests that in the case of $A7_{PRD}$ fibrillization, the primary nucleation process is partially dependent on initial monomer concentration, whereas the secondary process of fibril propagation is independent of monomer concentration.

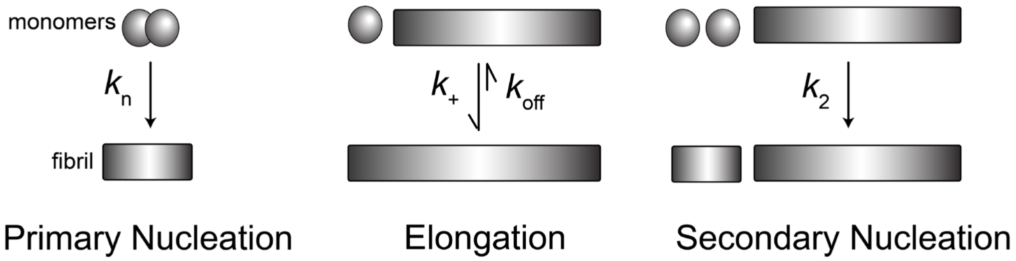

Figure S11. Microscopic processes involved in protein fibrillization, and the associated rate constants. k_n is the rate constant for the formation of primary nuclei, k_+ is the fibril elongation rate constant, and k_2 is the rate of formation of secondary nuclei, adapted from Meisl et al.[15]

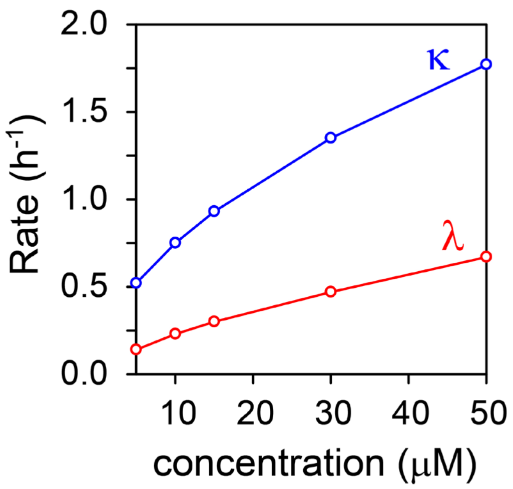

Figure S12. Comparison of the rates of the primary and secondary processes, λ and κ, respectively, involved in fibrillization of A7PRD. The rates were calculated as follows: $\lambda = [2 \times k_n k_+ \times M_0^{nc}]^{1/2}$ and $\kappa = [2 \times k_2 k_+ \times M_0^{n2+1}]^{1/2}$; see Fig. S11 for the description of rate constants, namely k_n, k_+ and k_2. n_c and n_2 represent the sizes of primary and secondary nuclei, respectively. M_0 represents the starting concentration of A7PRD monomer. The values obtained from global fit of ThT data (raw data is shown in Fig. S9, and fits are shown in Fig. 3I, main text) using the program Amylofit[15] were: $n_c = 1.3$, $n_2 = 0.1$, $k_n k_+ = 1.3e{+}5\ M^{-1.3}h^{-2}$ and $6.3e{+}4\ M^{-0.1}h^{-2}$.

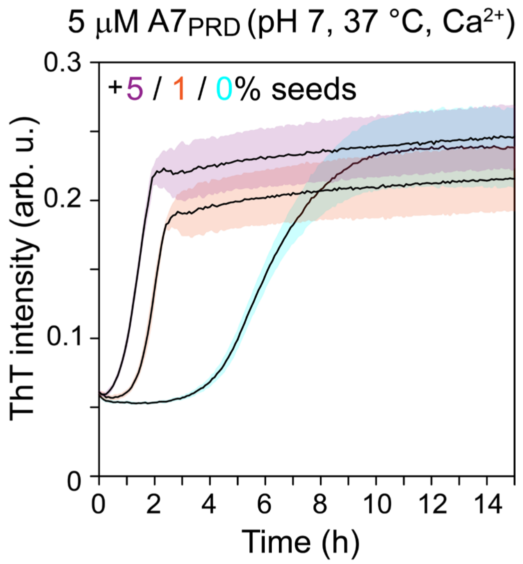

Figure S13. The effect of seeding on the aggregation kinetics of A7$_{PRD}$. Fibrillization of 5 µM A7$_{PRD}$ in the presence of varying amounts of A7$_{PRD}$ fibril seeds (0, 1, and 5%), $n = 3$, mean (solid line), SD (shaded region). All measurements were carried out at 37 °C under non-agitated conditions. The buffer composition as follows: 25 mM HEPES, pH 7, 5 mM calcium, and 20 µM ThT.

Table S1. Recombinant constructs used in current study[a,b].

Construct	Addgene accession no.	Competenet cells	Induction temperature	Yield[c] (mg/L)
A7	198635	BL(21)DE3	16 °C	15 mg/L
$A7_{PRD}$	190066	BL(21)DE3	16 °C	30 mg/L
$A7_{PRD}^{Strep}$	190067	BL(21)DE3	16 °C	25 mg/L

a) BL21(DE3) cells were obtained from Agilent (catalog no. 200131).

b) Cultures were grown in LB media overnight upon induction with 1 mM IPTG. LB capsules were obtained from MP Biomedicals (catalog no. 3002-036), and were used according to the manufacturers' protocols.

c) The yield represents the total amount of TEV-cleaved protein obtained from a liter of bacterial culture.

References.

(1) Schmidt, T. G. M.; Batz, L.; Bonet, L.; Carl, U.; Holzapfel, G.; Kiem, K.; Matulewicz, K.; Niermeier, D.; Schuchardt, I.; Stanar, K., Development of the Twin-Strep-tag and its application for purification of recombinant proteins from cell culture supernatants. *Protein Expr. Purif.* **2013,** *92* (1), 54-61.

(2) Huth, J. R.; Bewley, C. A.; Jackson, B. M.; Hinnebusch, A. G.; Clore, G. M.; Gronenborn, A. M., Design of an expression system for detecting folded protein domains and mapping macromolecular interactions by NMR. *Protein Sci.* **1997,** *6* (11), 2359-64.

(3) Ramaraju, B.; Nelson, S. L.; Zheng, W.; Ghirlando, R.; Deshmukh, L., Quantitative NMR study of insulin-degrading enzyme using amyloid-beta and HIV-1 p6 elucidates its chaperone activity. *Biochemistry* **2021,** *60* (33), 2519-2523.

(4) Elias, R. D.; Ramaraju, B.; Deshmukh, L., Mechanistic roles of tyrosine phosphorylation in reversible amyloids, autoinhibition, and endosomal membrane association of ALIX. *J. Biol. Chem.* **2021,** *297* (5), 101328.

(5) Elias, R. D.; Ma, W.; Ghirlando, R.; Schwieters, C. D.; Reddy, V. S.; Deshmukh, L., Proline-rich domain of human ALIX contains multiple TSG101-UEV interaction sites and forms phosphorylation-mediated reversible amyloids. *Proc. Natl. Acad. Sci. U.S.A.* **2020,** *117* (39), 24274-24284.

(6) Zhao, H.; Brautigam, C. A.; Ghirlando, R.; Schuck, P., Overview of current methods in sedimentation velocity and sedimentation equilibrium analytical ultracentrifugation. *Curr. Protoc. Protein Sci.* **2013,** *Chapter 20*, Unit20.12.

(7) Deshmukh, L.; Schwieters, C. D.; Grishaev, A.; Ghirlando, R.; Baber, J. L.; Clore, G. M., Structure and dynamics of full-length HIV-1 capsid protein in solution. *J. Am. Chem. Soc.* **2013,** *135* (43), 16133-47.

(8) Deshmukh, L.; Ghirlando, R.; Clore, G. M., Investigation of the structure and dynamics of the capsid-spacer peptide 1-nucleocapsid fragment of the HIV-1 Gag polyprotein by solution NMR spectroscopy. *Angew. Chem. Int. Ed. Engl.* **2014,** *53* (4), 1025-8.

(9) Deshmukh, L.; Ghirlando, R.; Clore, G. M., Conformation and dynamics of the Gag polyprotein of the human immunodeficiency virus 1 studied by NMR spectroscopy. *Proc. Natl. Acad. Sci. U. S. A.* **2015,** *112* (11), 3374-9.

(10) Elias, R. D.; Zhu, Y.; Su, Q.; Ghirlando, R.; Zhang, J.; Deshmukh, L., Reversible phase separation of ESCRT protein ALIX through tyrosine phosphorylation. *Sci. Adv.* **2023,** *9* (28), eadg3913.

(11) Nelson, S. L.; Li, Y.; Chen, Y.; Deshmukh, L., Avidity-based method for the efficient generation of monoubiquitinated recombinant proteins. *J. Am. Chem. Soc.* **2023,** *145* (14), 7748-7752.

(12) Shihora, A.; Elias, R. D.; Hammond, J. A.; Ghirlando, R.; Deshmukh, L., ALS variants of annexin A11's proline-rich domain impair its S100A6-mediated fibril dissolution. *ACS Chem. Neurosci.* **2023,** *14* (15), 2583-2589.

(13) Gidi, Y.; Bayram, S.; Ablenas, C. J.; Blum, A. S.; Cosa, G., Efficient one-step PEG-silane passivation of glass surfaces for single-molecule fluorescence studies. *ACS Appl. Mater. Interfaces* **2018,** *10* (46), 39505-39511.

(14) Pautot, S.; Frisken, B. J.; Weitz, D. A., Production of unilamellar vesicles using an inverted emulsion. *Langmuir* **2003,** *19* (7), 2870-2879.

(15) Meisl, G.; Kirkegaard, J. B.; Arosio, P.; Michaels, T. C. T.; Vendruscolo, M.; Dobson, C. M.; Linse, S.; Knowles, T. P. J., Molecular mechanisms of protein aggregation from global fitting of kinetic models. *Nat. Protoc.* **2016,** *11* (2), 252-272.

(16) Holehouse, A. S.; Das, R. K.; Ahad, J. N.; Richardson, M. O. G.; Pappu, R. V., CIDER: Resources to analyze sequence-ensemble relationships of intrinsically disordered proteins. *Biophys. J.* **2017,** *112* (1), 16-21.

(17) Carey, J. L.; Guo, L., Liquid-liquid phase separation of TDP-43 and FUS in physiology and pathology of neurodegenerative diseases. *Front. Mol. Biosci.* **2022,** *9*.

(18) Dao, T. P.; Kolaitis, R. M.; Kim, H. J.; O'Donovan, K.; Martyniak, B.; Colicino, E.; Hehnly, H.; Taylor, J. P.; Castañeda, C. A., Ubiquitin modulates liquid-liquid phase separation of UBQLN2 via disruption of multivalent interactions. *Mol. Cell* **2018,** *69* (6), 965-978.e6.

(19) Ding, X.; Gu, S.; Xue, S.; Luo, S. Z., Disease-associated mutations affect TIA1 phase separation and aggregation in a proline-dependent manner. *Brain Res.* **2021,** *1768*, 147589.

(20) Zhang, X.; Vigers, M.; McCarty, J.; Rauch, J. N.; Fredrickson, G. H.; Wilson, M. Z.; Shea, J. E.; Han, S.; Kosik, K. S., The proline-rich domain promotes Tau liquid-liquid phase separation in cells. *J. Cell Biol.* **2020,** *219* (11).

(21) Ray, S.; Singh, N.; Kumar, R.; Patel, K.; Pandey, S.; Datta, D.; Mahato, J.; Panigrahi, R.; Navalkar, A.; Mehra, S.; Gadhe, L.; Chatterjee, D.; Sawner, A. S.; Maiti, S.; Bhatia, S.; Gerez, J. A.; Chowdhury, A.; Kumar, A.; Padinhateeri, R.; Riek, R.; Krishnamoorthy, G.; Maji, S. K., α-Synuclein aggregation nucleates through liquid–liquid phase separation. *Nat. Chem.* **2020,** *12* (8), 705-716.

(22) Shin, Y.; Brangwynne, C. P., Liquid phase condensation in cell physiology and disease. *Science* **2017,** *357* (6357), eaaf4382.

(23) Schmidt, T. G. M.; Skerra, A., The Strep-tag system for one-step purification and high-affinity detection or capturing of proteins. *Nat. Protoc.* **2007,** *2* (6), 1528-1535.
